# Serology of Lupus Erythematosus: Correlation between Immunopathological Features and Clinical Aspects

**DOI:** 10.1155/2014/321359

**Published:** 2014-02-06

**Authors:** Emanuele Cozzani, Massimo Drosera, Giulia Gasparini, Aurora Parodi

**Affiliations:** Di.S.Sal, Section of Dermatology, IRCCS Azienda Ospedaliera, Universitaria San Martino-IST, 16132 Genoa, Italy

## Abstract

Systemic lupus erythematosus (SLE) is an autoimmune disease characterized by the aberrant production of a broad and heterogenous group of autoantibodies. Even though the presence of autoantibodies in SLE has been known, for more than 60 years, still nowadays a great effort is being made to understand the pathogenetic, diagnostic, and prognostic meaning of such autoantibodies. Antibodies to ds-DNA are useful for the diagnosis of SLE, to monitor the disease activity, and correlate with renal and central nervous involvements. Anti-Sm antibodies are highly specific for SLE. Anti-nucleosome antibodies are an excellent marker for SLE and good predictors of flares in quiescent lupus. Anti-histone antibodies characterize drug-induced lupus, while anti-SSA/Ro and anti-SSB/La antibodies are associated with neonatal lupus erythematosus and photosensitivity. Anti-ribosomal P antibodies play a role in neuropsychiatric lupus, but their association with clinical manifestations is still unclear. Anti-phospholipid antibodies are associated with the anti-phospholipid syndrome, cerebral vascular disease, and neuropsychiatric lupus. Anti-C1q antibodies amplify glomerular injury, and the elevation of their titers may predict renal flares. Anti-RNP antibodies are a marker of Sharp's syndrome but can be found in SLE as well. Anti-PCNA antibodies are present in 5–10% of SLE patients especially those with arthritis and hypocomplementemia.

## 1. Introduction

Systemic lupus erythematosus (SLE) is an autoimmune disease characterized by the presence of autoreactive B and T cells, responsible for the aberrant production of a broad and heterogeneous group of autoantibodies (Table 1). Indeed, in 2004 Sherer et al. reported that one hundred sixteen autoantibodies have been described in SLE patients [1]. In SLE, especially in its systemic form (SLE), autoantibodies directed to nuclear (ANAs), cytoplasmtic, and cellular membrane antigens are considered the serological hallmark. ANAs consist of various types of autoantibodies characterized by different antigen specificities. These nuclear antigens include single strand (ss) and double strand (ds) DNA (deoxyribonucleic acid), histone proteins, nucleosome (histone-DNA complex), centromere proteins, and extractable nuclear antigens (ENA) (Smith antigen (Sm), Ro, La, ribonucleoprotein (RNP), etc.). ANAs are present in about 95% of SLE patients with an active disease. In patients with prevalent cutaneous lesions, ANAs have been found positive in 75% of cases.

Therefore, considering the very wide spectrum of discovered autoantibodies, the aim of the present paper is to highlight the most promising and significant ones from both immunopathologic and clinical perspectives.

The presence of autoantibodies in SLE was envisaged when lupus phenomenon was described by Hargraves et al. in 1948 [2] and then proven when it was understood that it was due to neutrophil phagocytosis of cell nuclei opsonised by autoantibodies. In 1957, antibodies to DNA were identified [3] and in 1966 Tan and Kunkel found autoantibodies directed to antigens different from DNA and described the anti-Sm antibodies [4].

Even though the presence of autoantibodies in SLE has been known for more than 60 years, still nowadays a great effort is being made to understand the pathogenetic, diagnostic, and prognostic meaning of such autoantibodies. In particular, studies have focused on ANAs, anti-C1q antibodies, and anti-phospholipid antibodies.

Demonstrating the pathogenic role of autoantibodies is an arduous task; nevertheless recent data from murine, and human models have clarified the key role of autoantibodies in severe organ involvements, such as nephritis and neuropsychiatric dysfunctions [5]. Common autoantibody-mediated mechanisms of damage in SLE include immune complex-mediate damage, cell surface binding and cytotoxicity, reactivity with autoantigens expressed on apoptotic or activated cell surface, penetration into living cells, and binding to cross-reactive extracellular molecules [6].

Beyond elucidating the mechanisms behind the disease, understanding the pathogenetic role of autoantibodies, might have therapeutic implications. Indeed, in a recent article Diamond et al., after discovering the antigenic specificity of a subset of anti-DNA antibodies, hypothesized a potential therapeutic strategy, using peptides to block the antigen-binding site of the pathogenetic antibody [7].

Pisetsky gives another extremely interesting perspective, based on different sources [8–10], on the role of ANAs in autoimmune diseases, hypothesizing a protective role of such antibodies [11]. ANAs would prevent the disease by inhibiting the immunological activity of nuclear antigens, promoting their clearance in a nonphlogistic way or blocking the formation of immune complexes. Indeed, in SLE anti-SSA/Ro and anti-SSB/La antibodies seem to exert a protective role from lupus nephritis [12]. This hypothesis requires further investigations but could translate into other interesting findings in SLE as well.

However, the biggest effort was made to understand the clinical implications of antibodies found in the sera of patients affected by SLE. Indeed, the diagnostic and prognostic values of such antibodies are well known and no less than two of the American College of Rheumatology (ACR) criteria for SLE [13] regard immunological abnormalities:

“10. Immunologic disorder:

1. Anti-DNA: antibody to native DNA in abnormal titer or
2. Anti-Sm: presence of antibody to Sm nuclear antigen or
3. Positive finding of antiphospholipid antibodies on:
  - An abnormal serum level of IgG or IgM anticardiolipin antibodies,
  - A positive test result for lupus anticoagulant using a standard method,
  - A false-positive test result for at least 6 months confirmed by treponema pallidum immobilization or fluorescent treponemal antibody absorption test

11. Positive antinuclear antibody: An abnormal titer of antinuclear antibody by immunofluorescence or an equivalent assay at any point in time and in the absence of drugs”

Many authors have recently questioned the validity of these criteria, for example, Bizzaro et al. demonstrated through a study of meta analysis that the anti-nucleosome antibodies (AnuA) test is superior for diagnostics than the test for anti-dsDNA antibodies [14]. Furthermore Doria et al. underline that the test for anti-ribosomal P protein antibodies has a sensitivity and specificity for the classification of SLE similar to that of anti-Sm antibodies and that it could possibly substitute anti-Sm antibodies in the ACR criteria [15, 16].

Furthermore, anti-ribosomal P protein antibodies correlate with the activity of the disease and are associated with neuropsychiatric manifestations of SLE, while anti-Sm antibodies are frequently static over the disease course and it is difficult to link them with clinical manifestations. Nevertheless, the Systemic Lupus International Collaborating Clinics (SLICC) group recently revised and validated the ACR SLE classification criteria, maintaining and further emphasizing the same immunological criteria [17]. Indeed, according to the SLICC rules, patients must satisfy at least 4 criteria, including at least one immunologic criterion, or the patient must have biopsy-proven lupus nephritis in the presence of antinuclear antibodies or anti-double stranded DNA antibodies.

ANAs can be useful to identify particular subsets of LE: Anti-dsDNA is associated with renal involvement, anti-Ro/SSA antibodies with photosensitive rash especially subacute lupus erythematosus (SCLE) as well as with serositis and haematological manifestations, anti-P ribosomal protein with neuropsychiatric disorders, and anti-RNP with arthritis, Raynaud's, and puffy fingers. In this regard, another interesting point of view is given by Shivastava and Khanna [18], who propose the cluster theory: according to which distinct autoantibody clustering correlates to particular clinical syndromes. Cluster 1 (anti-Sm and anti-RNP) is characterized by the lowest incidence of proteinuria, anaemia, lymphopenia, and thrombocythemia. Cluster 2 (anti-dsDNA, anti-Ro and anti-La) is associated with a higher rate of nephritic syndrome and leukopenia. Cluster 3 (anti-ds-DNA, LAC and aCL) is expectedly associated with thrombotic events [19]. Moreover, Ching et al. [20] studied the serological profiles of SLE patients, finding that most of them segregated into one of two distinct clusters defined by autoantibodies against Sm/anti-RNP or Ro/La autoantigens. The Sm/RNP cluster was associated with a higher prevalence of serositis in comparison to the Ro/La cluster.

## 2. Techniques

ANAs can be detected by various assays: indirect immunofluorescence (IIF) using cultured cells as substrates, enzyme-linked immunosorbent assay (ELISA), and farr radioimmunoassay (RIA).

IIF and ELISA are most popular in routine work. ELISA is more sensitive but less specific while IIF is sensitive, reproducible, and easy to perform. ELISA is preferable when the exact titration of ANAs is needed in the follow-up of SLE.

Lately, multiplexed ELISA assays have been used for ANAs titration and these new sophisticated techniques are able to detect simultaneously multiple autoantibodies from a single sample. Until now, various studies report overall agreement between the detection of lupus autoantibodies by conventional ELISA and by multiplexed ELISA assays [46–48].

Monolayer of cultured cells, particularly HEp2 (a human laryngeal carcinoma cell line), is now considered the gold standard for IIF. In cultured cells used for IIF, the antigens are in the native location and form, undenatured or minimally denatured, and the nuclei and nucleoli are clearly visible in dividing cells.

**Figure 1:**
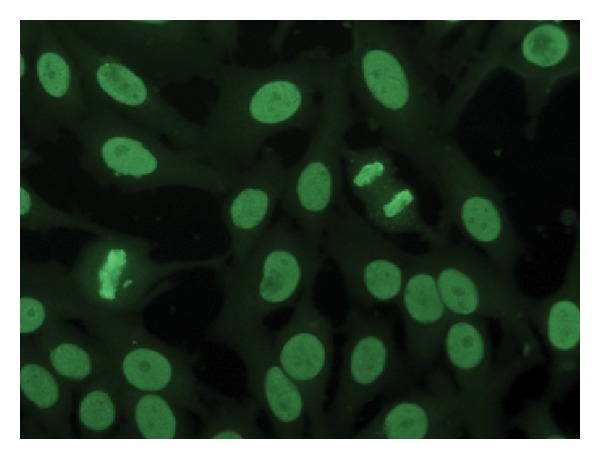
IIF on Hep2 cells: homogeneous pattern. Dilution 1 : 40.

About 40 different fluoroscopic patterns have been described in IIF, related to different antibody specificities. The most common are homogeneous, peripheral or ring, speckled, nucleolar, pleomorphic speckled, nuclear dots, and nuclear membrane. Generally, the homogenous pattern is linked to SLE (Figure 1). ANA pattern has some correlation with clinical subsets, such as a shrunken peripheral pattern with renal disease, a fine particulate pattern in SCLE, and a homogeneous pattern with anti-histone antibodies [49]. However, the homogenous pattern can be found in many other autoimmune diseases and, in contrast, various ANA patterns may coexist in the same disease. For these reasons, more specific tests, such as the anti-dsDNA test or anti-ENA test are necessary for a precise diagnosis, according to the well-known “cascade testing” as suggested by guidelines [50]. Indeed, it must be kept in mind that ANAs may be found not only in autoimmune diseases, but also hepatic diseases, malignancies, chronic infections, thyroid diseases, and even in individuals with no medical condition, particularly elderly people [51, 52].

Ippolito et al. [53] report the results of current serologic tests for SLE are generally consistent with the historical ones. However, probably due to their better sensitivity, current serological tests yield a certain percentage of additional positives. Further, due to a lower sensitivity in the past tests for C3 and C4 detected more frequently the depletion of these factors.

## 3. Anti-DNA Antibodies

Anti-DNA antibodies constitute a subgroup of antinuclear antibodies that bind to either single-stranded or double-stranded DNA [54]. Both subtypes of DNA-binding antibodies may be found in SLE. Nonetheless, while some authors highlighted a possible role of anti-ssDNA antibodies in the diagnosis and follow-up of SLE, especially when anti-dsDNA antibodies were negative [55, 56], others doubted the specificity and utility of this test [57–60]. Instead, because of their high specificity, anti-dsDNA antibodies are universally used as a diagnostic criterion for SLE (70–98% of patients are positive for such antibodies) [12] and for monitoring the clinical course of the patient [61] (every 6 weeks, for example), especially in the presence of an immunosuppressive treatment that reduces their production. IIF on *Crithidia luciliae* (Figure 1), RIA, and ELISA is the most commonly used assays to detect anti-dsDNA antibodies. IIF-based *Crithidia* assay is probably the most specific technique, but ELISA is the most practical and clinically relevant method. In IIF anti-dsDNA antibodies correlate with a shrunken peripheral ANA pattern [49]. It is generally accepted that anti-dsDNA antibodies, in particular of the IgG isotype, have an important pathogenetic role in SLE. A clear-cut relationship exists, for example, between anti-dsDNA antibodies (R4A antibody) [7] and disease activity in nephritis [62]. Anti-DNA-DNA immune complexes can deposit in the mesangial matrix and their subsequent complement activation leads to inflammation and mesangial nephritis. Moreover, anti-dsDNA antibodies also contribute to the end-stage lupus nephritis by directly binding exposed chromatine fragments in glomerular basement membrane [5]. On the other hand, IgM-class anti-dsDNA antibodies seem to have a protective role for nephropathy [63, 64]. Furthermore De Giorgio et al. demonstrated that a subset of anti-DNA antibodies cross-reacts with N-methyl-D-aspartate receptors (NMDAR), and through an excitotoxic mechanism, could induce neuronal apoptosis. Anti-NMDAR antibodies are present in 40% of lupus patients and some reports have supported the correlation between such antibodies and the presence of neuropsychiatric lupus [23, 65, 66], while others have not [67]. More recently, Franchin et al. have demonstrated that anti-NMDAR antibodies also bind C1q; therefore, they hypothesized that this subset of anti-DNA antibodies contributes in lupus pathogenesis through direct targeting of C1q on glomeruli and also through removal of soluble C1q thereby limiting the ability of C1q to suppressor of immune activation [68].

## 4. Anti-Sm Antibodies

Sm antigen consists of at least 4 proteins: B (28 kDa), B1 (29 kDa), D (19 kDa), and E (13 kDa). Anti-Sm antibodies are a highly specific marker for SLE and Anti-Sm reactivity is not described in other diseases. Their sensitivity is however low. In fact, anti-Sm antibodies are detectable only in 20% of SLE white patients, but 30–40% in black and Asian people. Clinical correlations of these autoantibodies remain unclear [12] and generally show persistent expression over time [11]. In some studies anti-Sm titers were found to fluctuate with disease activity and treatment [69], but it is unclear whether serial monitoring predicts relapse [70].

## 5. Anti-Nucleosome Antibodies

The antigen consists of pairs of 4 core histones: H2A, H2B, H3, and H4, forming the histone octamer around which 200 pairs of basis of DNA are wound twice, with H1 bound on the outside. Anti-nucleosomes antibodies (ANuA) react exclusively to nucleosomes and not to individual histones or native non-protein-complexed DNA [71].

**Figure 2:**
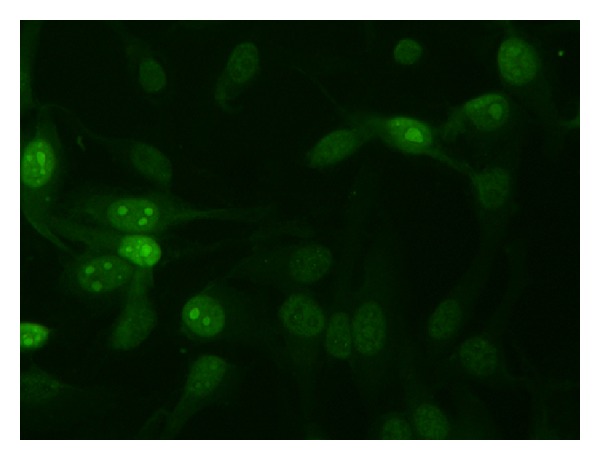
IIF on HEp2 cells: speckeld and nuclear and nucleolar staining (anti-SSA/Ro antibodies). Dilution 1 : 40.

Although anti-nucleosome antibodies can be seen in IIF as homogeneous pattern (Figure 2), only ELISA detects them.

They represent the first serological marker of SLE described and, at present, nucleosomes are considered a major autoantigen in SLE in which they are positive in about 85% of patients and probably play an important pathogenetic role [29]. There is major evidence that nucleosome antibodies play an important role in the pathogenesis of SLE, being the first ones to appear in murine lupus models before the onset of any other autoantibodies, which are only later produced by B cells, stimulated by nucleosome-specific T cells through epitope spreading [72]. In glomerulonephritis, nucleosomes facilitate binding of autoantibodies to glomerular basement membranes with an increased permeability and inflammatory response [5, 73].

According to Bizzaro's meta-analysis ANuA test appears to have an adequate level of diagnostic accuracy for SLE, with equal specificity, but higher sensitivity, positive likelihood ratio, and diagnostic odds ratio than anti-dsDNA antibodies test [14]. Indeed, they could be one of the most sensitive markers in the diagnosis of SLE, especially in anti-ds-DNA-negative patients [74]. Furthermore, there is a strong correlation between the level of anti-nucleosome antibodies and lupus disease severity [23, 75, 76]. ANuAs are probably better to predict flares in quiescent lupus [77].

## 6. Anti-Histone Antibodies

The target antigens are 5 major classes of histones (H1, H2A, H2B, H3, and H4), which organize and constrain the topology of DNA.

ELISA is the only reliable method for detection of anti-histone antibodies. It is important to use IgG-specific antibodies and not IgM that are not specifically related to the disease. Using IIF on standard substrates anti-histone antibodies produces a homogeneous, chromosome-positive staining of the nucleus.

These autoantibodies are characteristic of particular subset of SLE. In fact, anti-H2A-H2b antibodies are a sensitive test in drug-induced SLE. About 96% of patients with SLE induced by procainamide [31] and 100% of patients with SLE induced by penicillamine, isoniazid [32], and methyldopa have anti-histone antibodies. Nonetheless, they are also present in idiopathic SLE (70% of patients with SLE [12]), in rheumatoid arthritis, Felty's syndrome, Sjögren's syndrome (SS), systemic sclerosis (SSc) [78], primary biliary cirrhosis, infectious diseases (including HIV infection), and even neurological disorders such as Alzheimer's disease and dementia. In our experience, anti-histone antibodies are found in 10% of SLE patients and in 40% of SSc patients [79]. However, because of their low specificity these anti-histone antibodies albeit more prevalent, are not pathognomonic of drug-induce SLE [80]. This apparent paradox might be explained by the fact that the metabolites of offending drugs probably have the capacity to disrupt nonspecifically central immune tolerance to chromatin [81]. From a pathogenetic point of view, the histone-anti-histone antibody system might play a role in the perpetuation of murine lupus nephritis [82] and recently Sui et al. demonstrate in their study a strong association between simultaneous positivity to anti-DNA, anti-nucleosome, and anti-histone antibodies and renal disease activities, especially in proliferative glomerulonephritis [83].

## 7. Anti-SSA/Ro Antibodies

SSA/Ro antigen is a ribonucleoprotein containing small uridine-rich nucleic acids known as hY1, hY3, hY4, and hY5 (hY is the abbreviation of human cytoplasmic). SSA/Ro antigen consists of at least of 4 proteins: 45, 52, 54, and 60 kDa, respectively, with the best known of them being the 52 and 60 kDa proteins [84].

The most sensitive and specific method for detection of anti-SSA/Ro antibodies is ELISA. Using tumoral cell lines transfected with SSA/Ro antigen (HEp 2000) as a substrate, IIF is useful too, showing a typical speckled nuclear and nucleolar staining (Figure 2).

Anti-SSA/Ro antibodies might have a pathogenetic role in the initiation of tissue damage especially in photosensitive SLE, for ultraviolet radiation has been shown to induce *de novo* synthesis and the expression on the cell surface of SSA/Ro polypeptides in keratinocytes [85, 86].

Since the 1980s, it was known that anti-SSA/Ro and anti-SSB/La antibodies can cross the maternal placenta and determine neonatal lupus erythematosus (NLE). Indeed, anti-SSA/Ro as well as also anti-SSB/La antibodies bind to fetal heart conduction tissue and inhibit cardiac repolarization [87], determining isolated complete atrioventricular block (CHB). Other frequently observed manifestations of NLE are cutaneous rash, haematological disorders (thrombocytopenia, anemia, and leukopenia), and liver dysfunction [88], all of which tend to resolve within the time of clearance of maternal antibodies from the infant's circulation.

In a recent paper, it was reported that newborns from mothers with high to moderate titers of anti-SSA/Ro antibodies are more likely to develop cardiac manifestations of NLE, independently from the anti-SSB/La titers, while infants with prenatal exposure to high titers of anti-SSB/La antibodies were most likely to present non-cardiac manifestations [89].

Anti-SSA/Ro antibodies can be detected in 70–100% of patients with SS, in 30–70% of patients in particular in SCLE and NLE (70–80%) and with a lower frequency also in discoid LE (5–20%). Antibodies to the 52 kDa subunit are more specific for SS while antibodies to the 60 kDa subunit are more frequent in SLE patients. Anti-Ro and Anti-La antibodies are found earlier than other SLE-related autoantibodies and are present on average 6.6 years before the the diagnosis of SLE [33]. A close association between anti-SSA/Ro antibodies and late onset of SLE (average age of 50) was suggested [34]. Anti-SSA/Ro antibodies correlate with photosensitivity, SCLE, cutaneous vasculitis (palpable purpura), and haematological disorders (anemia, leukopenia, and thrombocytopenia) [35, 90–92].

There are discordant data regarding the association between anti-SSA/Ro titers and the disease activity, but it seems that anti-SSA/Ro antibody levels tend to decline when patients are treated with cytotoxic drugs [93–97].

Recently, greater attention is being paid toward distinguishing the two subtypes of anti-SSA/Ro: anti-SSA/Ro60 and anti-Ro52/TRIM21. A recent retrospective study conducted by Menendez et al. supports their routine distinction in clinical practice, since the two subtypes show different associations with different clinical subtypes of SLE. Indeed, anti-SSA/Ro60 are more frequently reported in association with SLE and CLE. Nevertheless, the pattern with both anti-SSA/Ro60 and anti-Ro52/TRIM21 is more frequent in SCLE and anti-Ro52/TRIM21 is more strongly associated with CHB [98]. In particular, the antibodies that seem to be strictly linked to CHB are directed against peptide aa 200–239 of subunit 52 kDa of Ro/SSA antigen [99].

## 8. Anti-SSB/La Antibodies

The SSB/La particle is a 48–50 kDa nuclear phosphoprotein composed of 2 distinct regions of 28 and 23 kDa [100]. The larger domain contains a RNA binding site that binds RNA polymerase III transcripts. Although anti-SSB/La antibodies were originally detected by immunodiffusion and counterimmunoelectrophoresis, they are now commonly detected by ELISA and immunoblotting.

Even though there is no direct evidence of a pathogenetic role of anti-SSB/La antibodies in SS and SLE, their presence in maternal blood is strongly associated with NLE and congenital heart block. In fact, both SSB/La and SSA/Ro antibodies bind to the surface of the fibres of the heart suggesting that the maternal anti-SSB/La and anti-SSA/Ro antibodies bind to the surface of cardiac muscle cells and damage them. Anti-SSB/La antibodies are the serological marker of SS [101]: if detected by ELISA, anti-SSB/La antibodies are present in 90% of patients with primary SS and 50% with secondary SS. In SLE, anti-SSB/La antibodies are instead present only in about 10% of patients with lower prevalence of renal disease. About 30% of patients with SCLE have anti-SSB/La antibodies.

## 9. Anti-Ribosomal P Antibodies

Ribosomes are complex macromolecular structures incorporating both protein and ribonucleic acid (RNA) elements. Mammalian ribosomes are formed by the 60S and 40S subunit. The 40S subunit is a ribonucleoprotein complex containing a single 18S species of RNA and 33 different basic proteins. The 60S subunit incorporates 3 distinct species of RNA, 46 different basic proteins, and 3 phosphoproteins named P0, P1, and P2 of 38, 19 and 17 kDa, respectively, that are the most important antigen targets of anti-ribosomal antibodies [102].

The specificity of autoantibodies directed against ribosomal components is evaluated by immunoblotting, but their presence is already suggested in IIF by a cytoplasmatic pattern. In the routine work, however, they are usually detected by ELISA. In comparative studies immunoblotting and ELISA seem to give the same diagnostic accuracy [103]. More recently, the international multicentre evaluation of the clinical accuracy of a new ELISA based on recombinant P polypeptides demonstrated that a combination of all three P proteins resembling the native heterocomplex P0 (P1/P2)_2_ as antigen gives the best accuracy [104].

Anti-ribosomal P antibodies seem to have an intriguing pathogenetic potential that needs further investigations. Indeed, anti-ribosomal P antibodies may exert different cellular effects by binding to the surface of T cells, monocytes, and endothelial cells [21].

They are able to penetrate into living cells by binding a cell-surface 38 kDa protein, which is the corresponding surface version of P0 ribosomal protein. In this way, they can cause cellular dysfunction and tissue damage by inhibiting protein synthesis, inducing apoptosis or proinflammatory cytokine production [105]. More recently, two independent groups elucidated the neuropathogenic potential of anti-ribosamal P antibodies [106, 107]. Moreover, Caponi et al. demonstrated that anti-ribosomal P antibodies in some cases can cross react with cardiolipin, ssDNA, dsDNA, and also nucleosomes. Such data indicate a partial overlapping of anti-ribosmal P antibodies with the other autoantibody populations detected frequently in SLE. For this reason anti-ribosomal P might have a similar pathogenetic role, for instance, in NPSLE [108].

The autoimmune response to ribosomal components is quite specific for SLE. Anti-ribosomal P antibodies occur in 13–20% of Caucasian SLE patients and in more than 40% of Asian patients [37].

Since the first prospective study in 1987 by Bonfa et al. [52] reporting a strong association between anti-ribosomal P antibodies and lupus psychosis, many other studies tried to confirm the utility of such antibodies in predicting NPSLE. However, the results were contrasting [38, 109]. Anyhow, many studies report associations with psychosis and especially depression.

## 10. Anti-Phospholipid Antibodies

The study of anti-phospholipid antibodies (aPL) antibodies began in 1906 when Wasserman introduced his serological test for syphilis [110]. In 1941, the active component was found to be a phospholipid, which was called cardiolipin [111]. After the 1950s, it became clear that people with positive Wasserman-test did not necessarily have syphilis but that they may have instead an autoimmune disorder, including SLE [112]. The term *lupus anticoagulant* (LAC) first used in 1972 should be abandoned because LA can be found in patients without SLE and it is associated with thrombosis and not with bleeding [113].

Anti-PL antibodies recognize a number of anionic negatively charged phospholipids, including cardiolipin (CL), LAC, phosphatidylserine (PS), phosphatidylinositol (PI), phosphatidylglycerol, and phosphatidic acid (PA). Neutrally charged autoantigen targets include phosphatidyl ethanolamine, phosphatidyl choline, platelet activating factor and sphingomyelin. These antibodies are usually detected with radioimmunoassay and ELISA. CL remains the most commonly used antigen for detecting anti-PL antibodies with ELISA. It is now clear, however, that the optimal binding of anti-PL antibodies depends on cofactors; the best known of them is termed Beta2-Glycoprotein I (Beta2GP1), that is, a 50 kDa B2 globulin involved in the regulation of blood coagulation [114]. ELISA testing for Beta2GP1 is also available [12].

As mentioned before, anti-PL antibodies are not confined to SLE patients but can be found in other autoimmune diseases, infections, malignant, and drug-induced disorders as well as in some apparently healthy individuals. In addition, anti-PL antibodies are positive in 30–40% of SLE patients, but only 1/3 of them develop clinical features of anti-PL syndrome, namely, venous thrombosis, arterial thrombosis, recurrent pregnancy loss, thrombocytopenia and haemolytic anaemia, *livedo reticularis,* and skin ulcers [39]. Furthermore, aPL antibodies are involved in cerebral vascular disease and are also implied in the pathogenesis of focal damage in NPSLE. In particular, anti-beta2GPI antibodies are the most thrombogenic and may exert a pathogenetic potential either as a strong procoagulant factor in the cerebral circulation or by directly interacting with neuronal tissue [5].

## 11. Anti-C1q Antibodies

C1q is a cationic glycoprotein of 410–450 kDa, which binds to the Fc portions of immunoglobulins in immune complexes to initiate complement activation via the classical pathway [115]. C1q is produced by macrophages, monocytes, dendritic cells, fibroblasts, and epithelial cells and acts like a binding molecule between debris from cellular apoptosis (apoptotic blebs) and macrophages. Therefore, anti-C1q antibody development seems to be related to a deficiency in apoptotic cell clearance, as suggested by the fact that such antibodies from SLE patients specifically bind to C1q on apoptotic cells [116].

Anti-C1q antibodies are commonly detected by ELISA. From a pathogenic point of view anti-C1q antibodies probably amplify glomerular injury but only when C1q has already been brought to the site by other types of glomerular-reactive autoantibodies [117]. Furthermore, Hegazy et al. recently reported in their study a strong correlation between anti-C1q antibodies and cutaneous lupus and hypothesised a potential pathogenetic role in such context [40].

They are found in SLE with a prevalence ranging from 17% to 46%, especially in patients with nephritis [41]. Moroni suggests that the elevation of their titers may predict renal flares even better than anti-dsDNA antibody levels [118]. Elevated titres of anti-C1q antibodies are usually associated with the proliferative forms of lupus nephritis and with subendothelial deposits of immune complexes. They are therefore a useful marker for assessing both disease activity and progression of the renal disease [118]. Anti-C1q antibodies can be found also in other autoimmune diseases such as hypocomplementemic urticarial vasculitis syndrome, rheumatoid arthritis, Felty's syndrome, rheumatoid vasculitis, Sjögren's syndrome, membranoproliferative glomerulonephritis (MPGN), and IgA nephropathy [119, 120].

## 12. Anti-RNP Antibodies

**Figure 3:**
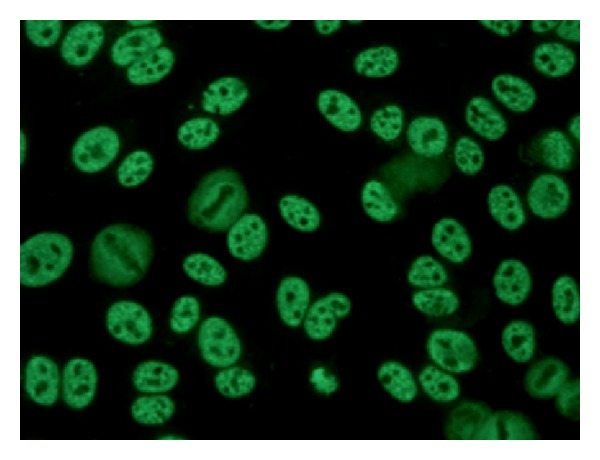
IIF on Hep2 cells: speckled pattern (anti-RNP antibodies). Dilution 1 : 40.

Anti-RNP antibodies are directed to at least 3 proteins of 70 kDa (U1), 33 kDa (protein A), and 22 kDa (protein C), respectively. In IIF anti-RNP antibodies produce a fine speckled staining (Figure 3). Anti-U1small nuclear (sn) RNP antibodies are considered pathognomonic for Sharp's syndrome (mixed connective tissue disease or MCTD), but they can be found in 20–30% of patients with SLE as well [42]. Their presence is associated with HLA DR4 and their prevalence is higher in African American patients [12]. Other diseases in which anti-U1snRNP activity is described include rheumatoid arthritis, polymyositis, SSc, and Sjögren's syndrome (SS). Data from recent experimental studies promote the hypothesis that U1snRNP antibodies participate in both innate and adaptive immune responses, implicating them in the pathogenesis of connective tissue disease [121]. According to some authors anti-RNP antibodies are more prevalent in patients with Raynaud's phenomenon and are associated with milder renal involvement [122]. Although, ultimately anti-U1 RNP antibodies do not reflect the disease activity and their utility in monitoring the latter remains unclear.

## 13. Anti-Proliferating Cell Nuclear Antigen (PCNA) Antibodies

**Figure 4:**
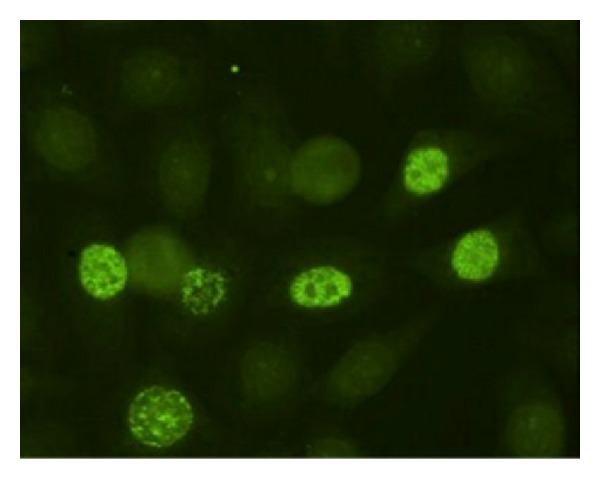
IIF on Hep2 cells: speckled pattern of varying intensity (anti-PCNA antibodies). Diluition 1 : 40.

Anti-PCNA antibodies can be detected by using IIF on cultured cells in which they show a characteristic nuclear speckled pattern of varying intensity (Figure 4). ELISA kits are also available. PCNA is an auxiliary protein for DNA polymerase delta. PCNA expression increases proportionally to DNA synthesis and/or cell growth, beginning in late G1, increasing in S, and decreasing in G2 cellular phases. Anti-PCNA antibodies are present in 5–10% of SLE patients especially those with arthritis and hypocomplementemia [44]. After treatment with steroids or cytotoxic drugs, anti-PCNA antibodies become undetectable.

## 14. Serology of SLE in Overlap Syndromes

SLE can be associated with other autoimmune diseases such as Sjögren's syndrome (SS), systemic sclerosis (SSc), rheumatoid arthritis (RA), dermatomyositis (DM)/polymyositis (PM), and determining overlap syndromes (OSs). OSs share clinical and immunological features of two or more distinct autoimmune diseases and might also have their own peculiar features. From a serological point of view OSs can be associated with a specific antibody profile (MCTD and SLE/SS) or not associated with a specific antibody profile (rhupus syndrome, SLE/SSc). MCTD has mixed features of SLE, SSc, DM/PM, and RA, in which anti-U1snRNP antibodies are the specific antibodies of the disease (see above). Anti-Ro/SS-A, anti-ssDNA, anti-Sm, anti-dsDNA [123], and anti-PL antibodies [124] have also been detected; nevertheless, they are not specific of MCTD. Recently, autoantibodies to angiotensin-converting enzyme 2 (ACE2) [125] were also reported in MCTD. SLE/SS patients have a higher frequency of SS-related immunological markers, such as rheumatoid factor (RF), polyclonal hypergammaglobulinemia, anti-Ro/SSA, and anti-La/SSB, while SLE-related antibodies are less frequent [126]. Anti-La/SSB antibodies are considered the serological markers of this OS. Most authors define rhupus syndrome as a condition characterized by signs and symptoms of both SLE and RA [127, 128]. In patients affected by such OS no specific antibody is identifiable and specific autoantibodies for SLE (anti-dsDNA and anti-Sm) and RA (anti-citrullinated peptides ACPA) coexist [126]. SLE/SSc overlap is a rare condition, in which a specific serological marker has not been identified yet, but a high incidence of anti-dsDNA and anti-Scl70 antibodies has been reported [126].

## 15. Conclusions

The comprehension of pathogenetic mechanisms is the starting point for the development of new and better laboratory tests, with various clinical implications. For example, the discovery of the cross-reactivity of certain types of anti-dsDNA antibodies with the NMDA receptor helped to comprehend the pathogenesis of NPSLE, but the detection of such antibodies in patients' sera could also be a potential predictive marker of the risk of developing NP disorders in SLE. Furthermore, distinguishing between the two different subtypes of anti-SSA/Ro antibodies might have interesting clinical implications. A better knowledge of the specificities of the antibodies might be a useful tool to subclassify patients with lupus and to predict which clinical manifestations they might develop. Detecting simultaneously a battery of various antibodies with multiplexed ELISA could be helpful for this purpose.

For the diagnosis of lupus certainly ds-DNA antibodies are an excellent biomarker, but we believe that perhaps ANuAs might be a better one, in accordance with Bizzaro's meta analysis, and considering that from a pathogenetic point of view these autoantibodies are the first ones to appear.

The role played by autoantibodies in the pathogenesis of lupus is yet to be revealed in many respects and the strive to find new and more valid biomarkers for a better management of the disease is constant, being lupus such a complex disease. Therefore, we believe there is still room for improvement as far as lupus serology is concerned.

## Figures and Tables

**Table 1:** Correlation between antibodies reactivity lupus subtypes and diagnostic utility.

Antibody	Target	Diagnostic utility	Associated lupus subtypes (prevalence)	Other associated diseases	References
ANAs	The cell nucleus	High sensitivity, but specificity is low	SLE (98%) LN (100%) MCTD Drug-induced lupus Discoid lupus (35%)	Hepatic diseases (autoimmune hepatitis A), malignancies, chronic infections, thyroid diseases, elderly people, SS, SSc, PM, DM, juvenile chronic arthritis, Felty's syndrome, relapsing polychondritis, and rheumatoid arthritis	[12, 21, 22]

Anti-dsDNA	Double strand DNA	High sensitivity and specificity for SLE. Correlate with disease activity	SLE (70–98%) LN (70%) NPSLE (44, 4–81, 6%)	RA, HIV and parvovirus B19 infections, myeloma, and type 1 autoimmune hepatitis	[12, 21, 23–26]

Anti-Sm	Sm^1^	Low sensitivity, but high specificity for SLE	SLE (20–40%) LN (14%) MCTD (8%)	EBV infections	[12, 21, 27, 28]

Anti-nucleosome	Nuclesome^2^	High sensitivity and specificity for SLE. Correlate with disease activity	SLE (61–85%) LN (60–90%) MTCD (41%)	RA, SSc, and SS	[14, 21, 29, 30]

Anti-histone	Histone	Low IgM Higher IgG	SLE (70%) LN (37%) Anti-H2A and Anti-H2b specific for SLE induced by drugs (96–100%)	Rheumatoid arthritis, SSc, PBC^3^, Alzheimer's disease, dementia, and infections	[12, 21, 31, 32]

Anti-SSA/Ro	SSA/Ro (proteins 60/52 kD)^4^	High prognostic value for NLE in pregnant women	SLE (30%) LN (31%) NLE (especially CHB) (90%) SCLE (70–80%) Discoid Lupus (5–20%)	SSc, IIM^5^, PBC, RA, and SS	[12, 21, 33–36]

Anti-SSB/La	SSB/La^6^	Moderate	SLE (10%) LN (14%) Protective for LN SCLE (30%) NLE (90%)	SS	[12, 21, 35, 36]

Anti-ribosomal P	Ribosomes^7^	Moderate	SLE (13–40%) LN (6%) NPSLE (especially psychosis and depression) (21%)	Hepatic diseases, malignancies, and RA	[12, 21, 37, 38]

Anti-phospholipid	Phospholipids^8^ (of cardiolipin, LACs are the most important ones)	High if Anti-PL syndrome is suspected	SLE (30–40%) LN (20–80%) Anti-PL syndrome: venous thrombosis, arterial thrombosis, recurrent pregnancy loss, thrombocytopenia and haemolytic anaemia, livedo reticularis, and skin ulcers.	Other autoimmune diseases, infections, malignancies, and drug-induced disorders, rheumatoid arthritis	[12, 21, 39]

Anti-C1q	C1q^9^	Low but useful to monitor evolution of LE nephritis	SLE (17–46%) LN (40–100%) CLE (44%)	Hypocomplementemic urticarial vasculitis syndrome, rheumatoid arthritis, and renal disease	[21, 40, 41]

Anti-RNP	RNP^10^	Unclear	SLE (20–30%) MCTD (100%)	Sharp's syndrome scleroderma, polymyositis, rheumatoid arthritis, SSc, Sj$\ddot{\text{o}}$gren's syndrome	[12, 42, 43]

Anti-PCNA	PCNA^11^	Low	SLE (5–10%)	Chronic hepatitis B and C	[44, 45]

^1^In biology, Sm proteins are a family of RNA-binding proteins found in virtually every cellular organism.

^2^A nucleosome is the basic unit of DNA packaging in eukaryotes, consisting of a segment of DNA wound in sequence around eight histone protein cores.

^3^Primary biliary cirrhosis.

^4^Ro60 is a ribonuclear protein containing small uridine-rich nucleic acids known. Protein 60 KD is located into the nucleus and nucleolus, while protein 52 is located into the cytoplasm.

^5^Idiopathic inflammatory myopathies.

^6^SSB/La particle is a 48–50 kDa nuclear phosphoprotein composed of 2 distinct regions of 28 and 23 kDa.

^7^P0, P1, and P2 of 38, 19, and 17 kDa, respectively, of the 60S subunit.

^8^Anionic phospholipis including cardiolipin (CL), LA, phophatidylserine (PS), phsphatidylinositol (PI), and phosphatidic acid (PA).

^9^C1q is a cationic glycoprotein of 410–450 kDa which binds to the Fc portions of immunoglobulins in immune complexes to initiate complement activation via the classical pathway.

^10^3 ribonucleoproteins: of 70 kDa (U1), 33 kDa (protein A), and 22 kDa (protein C), respectively.

^11^Anti-proliferating cell nuclear antigen.
